# Intermittent Hypoxia Induced Formation of “Endothelial Cell-Colony Forming Units (EC-CFUs)” Is Affected by ROS and Oxidative Stress

**DOI:** 10.3389/fneur.2018.00447

**Published:** 2018-06-14

**Authors:** Katia Avezov, Dror Aizenbud, Lena Lavie

**Affiliations:** ^1^The Lloyd Rigler Sleep Apnea Research Laboratory, Unit of Anatomy and Cell Biology, The Ruth and Bruce Rappaport Faculty of Medicine, Technion-Israel Institute of Technology, Haifa, Israel; ^2^Department of Orthodontics and Craniofacial Anomalies, The Ruth and Bruce Rappaport Faculty of Medicine, Technion-Israel Institute of Technology, Haifa, Israel

**Keywords:** intermittent hypoxia, obstructive sleep apnea, endothelial cell-colony forming units (EC-CFUs), oxidative-stress, N-acetylcysteine, NADPH oxidase inhibitors, endothelial tube formation

## Abstract

Intermittent hypoxia (IH)—the hallmark of obstructive sleep apnea (OSA)—increases leukocyte activation, production of NADPH-oxidase dependent reactive oxygen species (ROS) and oxidative stress, affecting endothelial function. However, IH and oxidative stress can also stimulate adaptive-protective mechanisms by inducing the development of Endothelial Cell-Colony Forming Units (EC-CFUs), which are considered as a good surrogate marker for endothelial progenitor cells (EPCs), and likely reflect a reparatory response to vascular damage or tissue ischemia by leukocytes. Blood samples were obtained from 15 healthy consenting volunteers to evaluate the effects of IH and sustained hypoxia (SH) *in vitro* on EC-CFUs development and functions. The variables measured included: their numbers, the area, the proliferative capacity and ROS production. Additionally, NADPH-oxidase, VEGF and nuclear factor-erythroid 2 related factor 2 (Nrf2) expression, as well as their paracrine effects on endothelial tube formation were determined. The involvement of ROS was probed using the anti-oxidant N-acetylcysteine (NAC) and NADPH-oxidase inhibitors apocynin and diphenyl-iodide. Compared to normoxia, IH-dependent increases in EC-CFUs numbers were observed, showing an individual donor-dependent trait. Also, the expression of VEGF and gp91phox, a subunit of NADPH-oxidase, were significantly increased. ROS production and oxidative stress markers were also significantly increased, but Nrf2 expression and colony size were unaffected by IH. Additionally, conditioned media harvested from IH- and SH-treated mature EC-CFUs, significantly increased endothelial tube formation. These effects were markedly attenuated or diminished by the ROS and NADPH-oxidase inhibitors employed. In conclusion, we show here for the first time that IH-associated oxidative stress promotes EC-CFUs' vascular and paracrine capacities through ROS. However, the large inter-individual variability expressed in EC-CFUs numbers and functions to a given IH stimulus, may represent an individual trait with a potential clinical significance.

## Introduction

Intermittent hypoxia (IH) is a common denominator in a wide range of pathological conditions including ischemic heart attacks and obstructive sleep apnea (OSA). The nightly IH associated with OSA leads to intermittent blood oxygen desaturation and sleep fragmentation inducing oxidative stress and inflammation. Consequently, powerful and complex effects on various biological systems are provoked, including activation and deactivation of vital signaling pathways (1). Moreover, OSA has an established association with various systemic pathological conditions. For instance, OSA patients are at an increased risk for cardiovascular diseases such as systemic hypertension, pulmonary hypertension, ischemic heart disease (IHD) and stroke (2, 3).

One of the underling mechanisms promoting the IH-induced damage to cells and tissues is through oxidative stress. OSA-associated IH was shown to cause influx of free radicals and reactive oxygen species (ROS) and is considered analogous to ischemia reperfusion injury (1, 4). In addition, leukocytes exposed to IH or in OSA are activated and their NADPH oxidase-dependent ROS production is increased (5). However, at the same time, OSA-associated IH may also stimulate adaptive-protective mechanisms (4).

Activation and mobilization of circulating endothelial progenitor cells (EPCs) was described as one of the adaptive-protective mechanisms in the cardiovascular system. EPCs can differentiate into mature endothelial cells and promote vascular repair. Yet, identification of EPCs by nomenclature is an ambiguous issue and there is a general lack of concordance in the stem cell field in which many distinct cell subtypes are continually grouped under the term “EPC” (6). One of the identification methods is based on the formation of colonies in cell cultures from peripheral blood. The most extensively used colony formation assay, described by Hill et al. (7), is that of blood derived endothelial cells-colony forming units (EC-CFUs), which originate from hematopoietic cell progenitors and have angiogenic and proliferative potential (7–9) These colonies, comprise of central cluster of round cells identified as angiogenic T cells, and are surrounded by spindle-shaped cells, predominantly derived from CD14+ monocytes and lymphocytes, yet they also express weak endothelial markers (10–12). These angiogenic T cells in the central cluster enhance the differentiation of EPCs *in vitro* and play an important role in new vessel formation *in vivo* (13). Moreover, the presence of T cells is essential for the formation of the EC-CFUs by CD14+ monocytes (14). Circulating levels of these angiogenic T cells are positively correlated with EC-CFUs numbers and inversely correlate with Framingham Risk Scores in humans (the Framingham Risk Score includes the following parameters: age, cholesterol, systolic blood pressure, hypertension, and cigarette smoking, and represents a 10-year risk for developing severe coronary heart disease). Therefore, circulating levels of angiogenic T cells may serve as a new biological marker for ischemic cardiovascular diseases (12). Also, EC-CFUs were shown to closely correlate with endothelial function and the combined Framingham Risk Score, and retain a measure of vascular health (7, 11, 13). Furthermore, the human EPCs expressing CD34+/AC133+/VEGF-R2+ were also identified as distinct primitive hematopoietic progenitors (15). Lambiase et al. (16) clearly demonstrated that coronary artery patients with higher EPCs numbers have higher collateralization, higher collateral flow index and higher tube formation, whereas the opposite pertains to patients with low EPCs numbers. Similarly, Matsuo et al. (17) demonstrated that higher EPCs numbers are correlated with higher collateralization and higher tube formation. Thus, although EC-CFUs are not a direct measure of EPCs, since they are closely correlated with EPCs numbers and vascular functions in cardiovascular patients *in vivo*, they are considered a good surrogate marker for EPCs (7, 12, 16–18). Collectively, it is currently believed that the development of EC-CFUs in culture likely reflects leukocyte activation as a reparatory response to vascular damage or tissue ischemia, indirectly contributing to tissue angiogenesis via paracrine mechanisms (10, 11). This is also corroborated by data demonstrating that various monocytes subsets are pluripotent and are modulated by environmental or local signals, inducing them to transdifferentiate into other cell types including endothelial-like cells (19, 20). Jointly, these findings suggest that angiogenic T cells and monocyte-derived EPCs are essential to the development EC-CFUs. The latter facilitating vasculo-protective capacities (13).

In a previous study from our laboratory, EPCs numbers, angiogenic T cells and monocyte VEGF expression were increased in the circulation of patients with acute myocardial infarction (AMI) and coexistent sleep disordered breathing (SDB) compared to patients with AMI but without SDB. Additionally, EC-CFUs numbers and their proliferative and angiogenic properties were also heightened in those patients. Similarly, the proliferative and angiogenic properties of EC-CFUs from healthy individuals were increased after exposure to IH *in vitro*, directly implicating the IH associated with OSA in altering EC-CFUs numbers and functions (12).

Given the apparent role of EC-CFUs in cardiovascular health and its association with OSA and IH on one hand, and the cardinal role that oxidative stress and ROS play in OSA associated cardiovascular co-morbidities, we sought to investigate whether ROS and oxidative stress are involved in EC-CFUs formation and their and paracrine-angiogenic capabilities on endothelial tube formation. We show here for the first time, that IH-associated oxidative stress promotes the formation of EC-CFUs', as well as their paracrine capacities through ROS. Yet, basal and IH-induced EC-CFUs numbers represent an individual trait.

## Materials and methods

### Blood donors' recruitment and sleep study

Blood samples were obtained from 15 (7 males/ 8 females) healthy non-smoker volunteers with a mean age of 26.6 ± 3 years and BMI of 23.7 ± 4.5 Kg/m^2^. Sleep studies were performed on all subjects using the WatchPAT-100 device (21), to rule out occult SDB. All subjects had less than 5 oxygen desaturation breathing events per hour (ODI) which is considered a normal value. The protocol was approved by the local Human Rights Committee of RAMBAM Medical Center according to the Declaration of Helsinki, and all participants signed an informed consent form. Some of the subjects were tested two to six times. All blood samples were withdrawn in the morning hours, collected into EDTA containing vacutainers (BD Plymouth, UK) and kept on ice until use.

### Cell culture of endothelial-cell colony forming units

Total mononuclear cells (MNCs), containing monocytes and lymphocytes, were isolated by density gradient centrifugation using LSM-lymphocyte separation medium (MP Biomedicals, USA), according to manufacturer's instructions. The recovered cells were washed twice with PBS and once in growth medium (Medium 199, Biological Industries, Israel) supplemented with 20% heat inactivated FCS, gentamycin (50 μg/ml) and amphoterycin-B (25 μg/ml). To eliminate possible contamination with macrophages, an initial pre-plating step in a 6-well plate using 3 × 10^6^ cells/well was performed. After 48 h, the non-adherent cells were collected and 1 × 10^6^ cells were re-plated onto new fibronectin coated 24-well plates for a final assessment of the number of colonies. Growth medium was replaced every 3 days, and the numbers of colonies were assessed 7 days after the initial plating. EC-CFUs were composed of central clusters of round cells surrounded by sprouting spindle-like cells as described in the literature (7, 12). EC-CFUs numbers per well were counted and averaged in 4 wells in each experiment using Axiovert 25 (Zeiss) light microscope at X10 magnification.

### *In vitro* intermittent (IH) and sustained hypoxia (SH) protocol

EC-CFUs were grown for 7 days as described above. On the day of re-plating, EC-CFUs were exposed to normoxia, IH, or SH in a custom-designed incubation chambers attached to an external O_2_-CO_2_-N_2_ computer driven controller using BioSpherix-OxyCycler-C42 system (Redfield, NY, USA). This system enables creating periodic changes in external O_2_ concentrations that control air gas levels in each chamber individually as previously described (22). Oxygen levels in the medium were determined by a fiber-optic dissolved oxygen electrode (BioSpherix, Redfield, NY, USA). The actual lowest % of O_2_ in the medium dropped to 5% during the hypoxic period for about 1.5 min, and this level of hypoxia was achieved after 15 min of incubation. In the reoxygenation period, O_2_ levels reached normoxic levels (20%) after 10 min of incubation. Carbon dioxide was held constant (5%) at all treatments. For modeling IH, EC-CFUs were exposed for 3 consecutive days (days 3–5 in culture) to 14 IH cycles/day (approximately 6 h/day). For SH, 5% continuous oxygen level was maintained in the medium for comparable times. Thereafter, the hypoxia treated cells were transferred to normoxia for additional 2 days until final maturation of the EC-CFUs (total 7 days in culture), after which various measures were performed. Control cells were maintained in normoxia for the entire period. In the cell proliferation assays with bromodeoxyuridine (BrdU) and NBT (nitro blue tetrazolium) test descr**i**bed below, EC-CFUs were cultured under Norm, IH and SH conditions for 5 consecutive days after re-plating (also total 7 days in culture). Of Note, exposing cells to IH or SH for three instead of 5 days for the development of colonies in culture and for endothelial tube formation was sufficient since similar EC-CFUs formation was observed under both time periods of exposure.

### Treatments of EC-CFUs with inhibitors

Following the stage of re-plating, EC-CFUs were exposed to Norm, IH, and SH for 3 days as described above, with or without various inhibitors. The following inhibitors were used: ROS scavenger—N-acetylcysteine (NAC) 1 mM, NADPH oxidase inhibitor—apocynin 100 μM, and NADPH oxidase inhibitor—diphenyl iodide (DPI) 5 μM (all purchased from Sigma-Aldrich). Each inhibitor was diluted with culture medium and was added 10 min prior to the various oxygen treatments. The inhibitors remained in the cell cultures throughout the treatments. For NBT test, 5 μM DPI were added to EC-CFUs cultures on the last day of the IH and SH cycles before the NBT test was performed.

### Nitro blue tetrazolium (NBT) test

The formation of ROS within the 7-day colonies was determined by NBT test (*n* = 3). The test depends on the direct reduction of NBT to an insoluble blue compound formazan by NADPH oxidase. Cytoplasmic clumps of formazan deposits are considered positive for ROS. The blue score positively correlates with cellular ROS production. NBT at 0.2%, (Sigma-Aldrich) was dissolved in RPMI 1640 without phenol red. Then, the cell culture medium was replaced with NBT containing medium and incubated at 37°C for 15 min. Subsequently, the cell cultures were washed and kept at room temperature for 10 min, then assessed by light microscopy. Experiments were carried out with and without DPI. DPI was added to EC-CFUs wells as specified in the inhibitors section Treatments of EC-CFUs With Inhibitors.

### Detection of protein carbonyls modifications

Protein carbonyl assay was used as an indicator of protein modification (per mg protein) following exposure to Norm, IH and SH. Commercially available OxyBlot Protein Oxidation Detection Kit (Millipore, USA), based upon 2,4-dinitrophenylhydrazine (DNPH) carbonyl derivation was used. The test was followed by immunodetection with western blot assay using anti-dinitrophenyl (DNP) antibodies and quantification by densitometry. Equal amounts of total protein (50 μg, determined by Bradford) were loaded onto each lane. The normoxia values for each subject served as a reference point. The amount of protein in each lane was also confirmed based on Ponceau S protein stain which is used to evaluate the total protein bands in each lane (23).

### Immunofluorescence analysis of NADPH oxidase subunits gp91-phox and p22phox, nuclear factor-erythroid 2 related factor 2 (Nrf2) and vascular endothelial growth factor (VEGF) in EC-CFUs

Specific fluorescence intensity (FI) of, gp91-phox, p22-phox Nrf2, and VEGF was detected using confocal laser scanning microscopy in EC-CFUs cultured on 13 mm diameter cover-slips under Norm, IH and SH and quantified by densitometry. EC-CFUs cultures were fixed as previously described (24) on ice-cold 4% paraformaldehyde for 15 min at room temperature, washed with PBS, incubated in medium containing 10% normal goat serum for 1 h at 37° C and permeabilized with 0.5% Triton X-100. Then the cells were incubated over-night with the specific primary antibodies diluted with blocking solution: anti- gp91-phox (1:150) (Abcam)/anti-cytochrome b-245 light chain (p22phox) (1:100) (BioLegend)/anti-Nrf2 (1:150) (Santa Cruz Biotechnology)/ anti-VEGF (1:250) (Abcam). Following incubation, the cells were washed with PBS and incubated for 1 h with secondary antibodies (1:400) Cy2-conjugated goat anti-rabbit IgG for gp91-phox, Nrf2, and VEGF and Cy5-conjugated goat anti-mouse IgG for p22phox (Jackson ImmunoResearch). After washing, cover slips were mounted on slides with mounting medium and DAPI for nuclear staining (Vectashield H-1000, Vector lab. Burlingame, CA). Slides were analyzed by a confocal laser scanning system (LSM 700) using Nikon E600 fluorescence microscope and Plan Apo x40 immersion oil objective. Fluorescent intensities were integrated with ImageJ (Wayne Rasband, NIH, USA).

### Determination of cellular proliferation within EC-CFUs

Five separate wells with cover slips were prepared for each oxygen condition (Norm, IH and SH, total 15 wells) at the re-plating step on day 3, and from day 3 to 7 an individual well in each condition was pulsed with bromodeoxyuridine (BrdU) (Sigma-Aldrich) at a 100 mmol concentration for 6 h, thereby providing a “snapshot” of the frequency of proliferation occurring for a 6 h period on each day in culture (10). After the last cycle on each day the supernatant was removed and colonies were fixed with ice-cold methanol for 10 min, washed with PBS and immersed in 2 M HCl solution for 2 h. Then the cells were washed 3 times with 0.1 M boric buffer and PBS before overnight incubation with anti-BrdU antibody (1:10) (Roche, Germany). Afterwards, the cells were stained with AEC staining mix kit (Sigma-Aldrich), washed with PBS and additionally stained with hemotoxylin, following by mounting with Immu-Mount mounting medium (Thermo, Pittsburg, PA, USA). Slides were analyzed with Olympus CX4 microscope at x10 magnification. Proliferation was expressed as the proportion of BrdU positive cells to EC-CFUs volume sum per well (cells/μm^3^). Colony volume (V) was calculated using the formula V = (πr^2^h)/3, where r = the radius of the base of the colony and h = the height of the colony, assumed to be equal to r (10).

### Endothelial tube formation assay with inhibitors

Endothelial tube formation is an *in vitro* assay for vascular function. On day 7 in culture, EC-CFUs conditioned media from Norm, IH and SH treatments were collected and centrifuged for 5 min at 300 × g to remove cell debris. Then, these conditioned media were used to determine endothelial tube formation. Flat-bottomed 48-well plates were coated with Extracellular Matrix (ECM) Gel (Sigma-Aldrich) (150 μl/well) and incubated for 30 min at 37° C to solidify. A total of 5 × 10^4^ EA.hy926 endothelial cells (ECs), generously provided by Edgell et al. (25), were cultured in 350 μl of conditioned media and incubated at 37° C for 24 h. Tube formation was monitored periodically under Axiovert 25 (Zeiss) light microscope (magnification X10), and after 24 h. Images were taken by AxioCam MRc (Zeiss) digital camera at the same magnification. Tube length per photographed field was measured by ImageJ program and averaged in 4 wells in each experiment. In addition, high-resolution whole-well imaging of the entire well of 24 well plate was photographed using Zeiss Axio observer microscope and high sensitive Hamamtsu Orca R2 camera. The automated image overlap feature of the instrument was used at 5% overlap. In this way the entire well, and the tube formation area was imaged. Post-acquisition, image files were automatically stitched, generating one image for each well. As a positive control for endothelial tube formation the complete endothelial growth medium EGM-2 (containing VEGF, human fibroblast growth factor (hFGF), insulin growth factor-1 (IGF-1), human epidermal growth factor (hEGF), hydrocortisone, ascorbic acid, GA-1000 and heparin) supplemented with 20% FCS was used. As a negative control, tube formation was carried out in DMEM medium supplemented with 20% FCS. Additionally, the effect of various inhibitors on endothelial tube formation was assessed using NAC at 1 mM, apocynin at 100 μM and DPI at 5 μM.

### Statistical analysis

Data was expressed as mean ± SD or ratios where appropriate. A two-tailed Student's *t*-test with Bonferroni correction was used for multiple comparisons. Therefore, in experiments with three variables, only values of ρ < 0.017 were considered significant. In experiments with six variables, values of ρ < 0.008 were considered significant. The SPSS 17 statistical package was used (IBM).

## Results

### Intermittent hypoxia affects EC-CFUs numbers in a donor dependent manner

**Figure 1:**
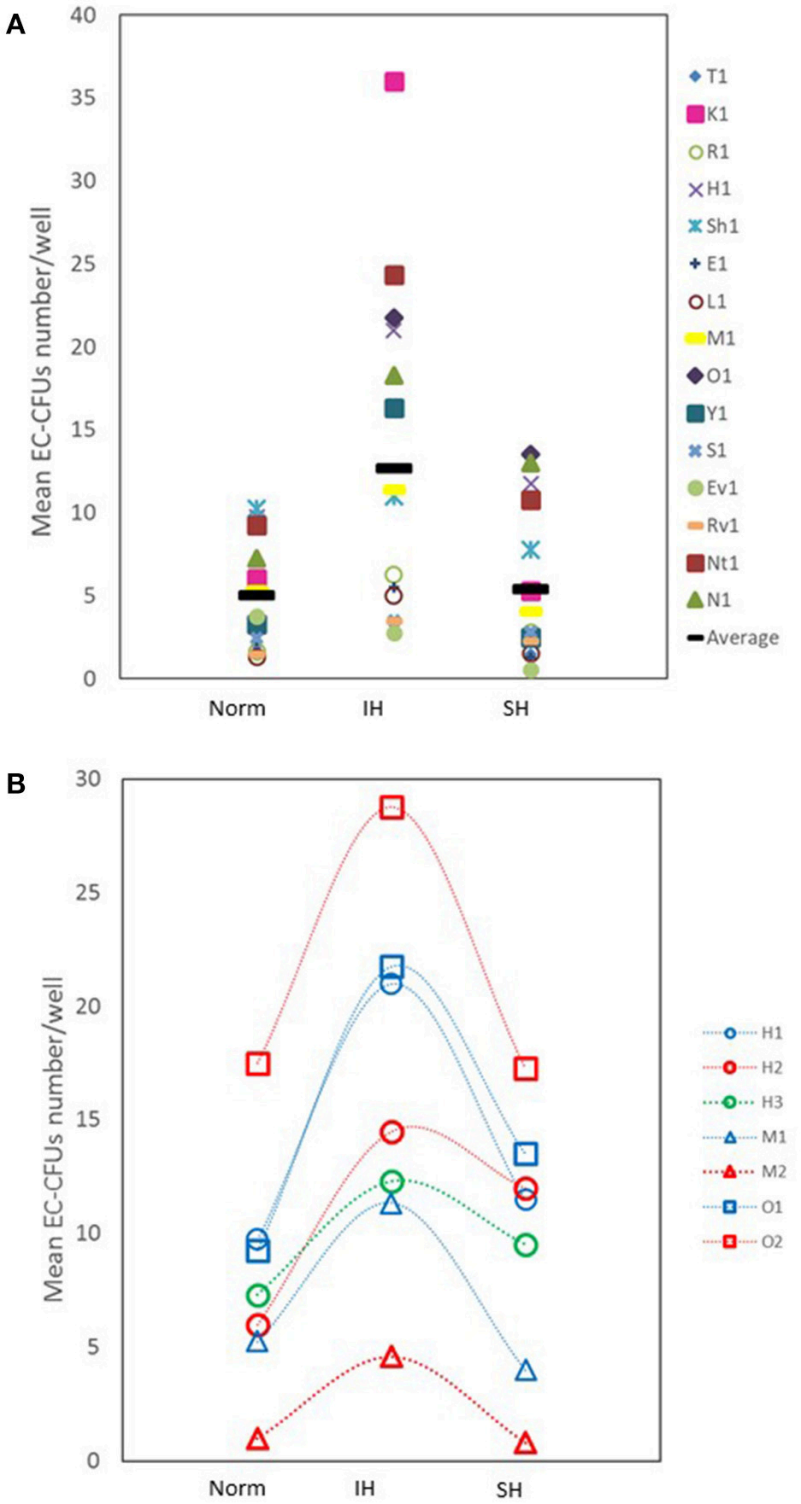
The effects of intermittent and sustained hypoxia, *in vitro*, on EC-CFUs numbers**. (A)** Individual and mean EC-CFUs numbers on the 7th day in culture (*n* = 15). The cells were exposed for three consecutive days to 14 intermittent hypoxia (IH) cycles, or to 6 h sustained (SH) hypoxia per day and compared to normoxia (Norm), as described in Methods. Each symbol represents a different donor. The horizontal bar represents the average value for each treatment. (IH 12.7 ± 10 vs. Norm 5 ± 3.3 EC-CFUs/well, *p* < 0.017; Norm vs. SH 5.4 ± 4.7 EC-CFU/well, *p* = NS). **(B)** EC-CFUs formation under Norm, IH and SH, in three donors who were tested two to three times. The first, the second, and the third (where available) tests were plotted (H1, patient H first test; H2, patient H second test; H3, patient H third test).

The number of EC-CFUs developed in culture was assessed after treating with Norm, IH and SH. Non-adherent cells (1 × 10^6^) from 15 healthy donors were re-plated after 48 h on fibronectin-coated 24 well plates and exposed to 14 IH cycles or to 6 h of SH daily, for 3 consecutive days in culture after replating, as described in Methods. Control plates were maintained in normoxia for comparable times. As depicted in Figure 1A, the mean number of colonies was significantly higher in cultures treated with IH as compared to normoxia (12.7 ± 10 EC-CFUs/well vs. 5 ± 3.3 EC-CFUs/well, *p* < 0.017). However, there were no significant differences between normoxia and SH-treated cultures (5.4 ± 4.7 EC-CFU/well).

Of note, three out of the 15 blood donors were studied 2-4 times, and the cells were cultured under Norm, IH and SH conditions as previously described. Figure 1B represents the EC-CFUs numbers that developed in culture from these donors and their response to Norm, IH and SH after testing each subject two to three times. Apparently, the cells from each donor maintained a similar response to the same stimulus on various experiments at all three oxygen conditions, while each of the donors maintained an individual donor-dependent response for EC-CFUs development in culture.

### Oxidative stress dependent EC-CFUs formation; effects of N-acetylcysteine (NAC) on EC-CFUs numbers

**Figure 2:**
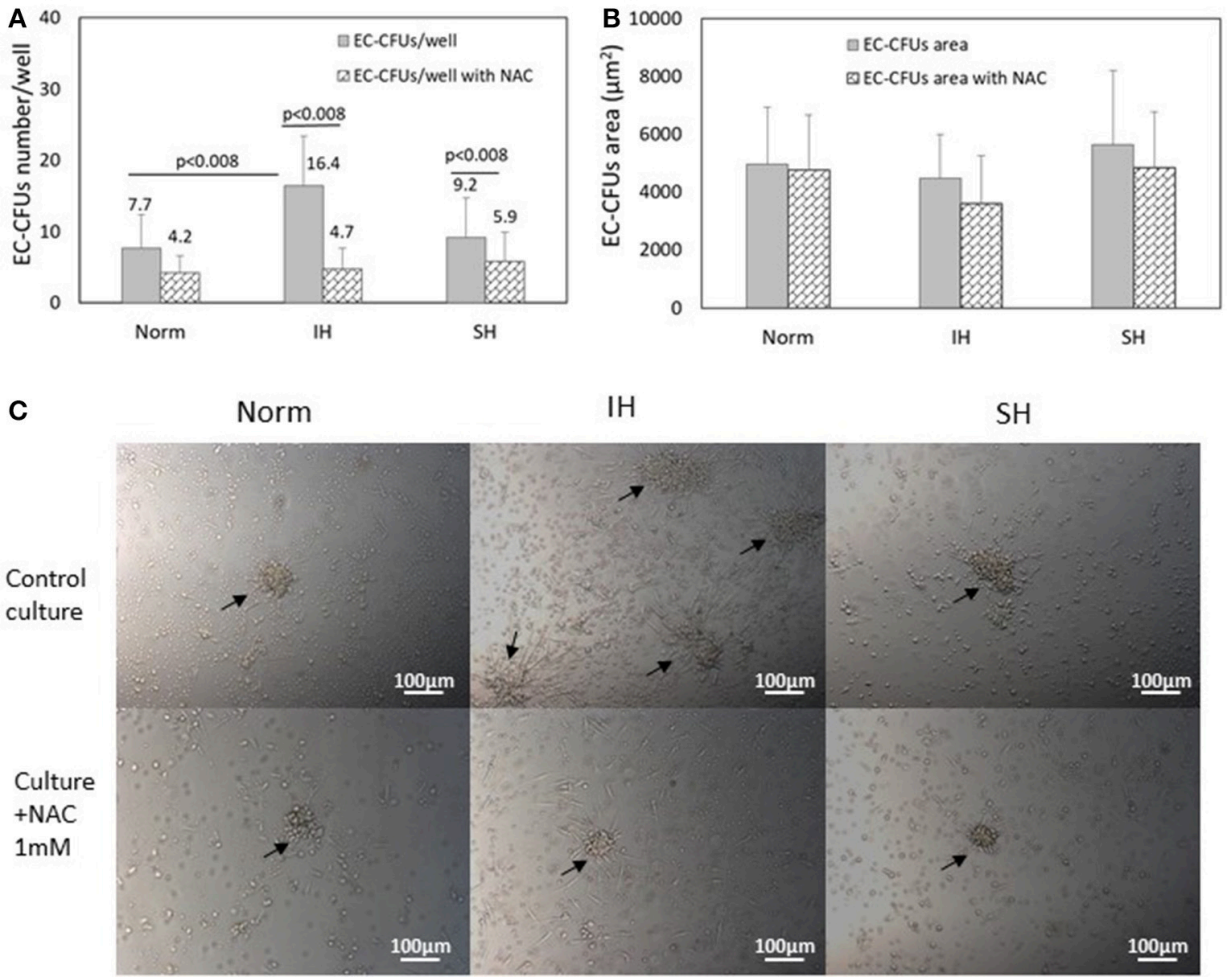
The effects of intermittent and sustained hypoxia, *in vitro* on EC-CFUs numbers and colony area with and without N-acetylcysteine. **(A)** Mean EC-CFUs numbers on the 7th day in culture with and without 1 mM N-acetylcysteine (NAC) (*n* = 9). The cells were exposed for 3 days to 14 intermittent hypoxia (IH) cycles per day (approximately 6 h/day) or to sustained hypoxia (SH) for an equal time per day and compared to normoxia (Norm), as described in Methods. In additional experiments, cells were exposed to 1 mM NAC concomitantly with Norm, IH and SH. (*p* < 0.008 for: Norm vs. IH; IH vs. IH+NAC; SH vs. SH+NAC). **(B)** Mean EC-CFUs size on the 7th day in culture with and without 1 mM NAC (*n* = 9) was unaffected. No significant differences were found between the areas of EC-CFUs treated with various oxygen treatments or with NAC. **(C)** Representative photomicrographs of EC-CFUs microscopic fields (X10) for Norm, IH and SH with and without 1 mM NAC. EC-CFUs colonies are shown by arrows.

Assessing the numbers of EC-CFUs which developed in cultures treated under Norm, IH and SH with and without the ROS scavenger N-acetylcysteine (1 mM) (*n* = 9) revealed that the mean number of colonies was significantly lowered in NAC treated cultures in all three oxygen conditions. However, the most striking decrease was noted under IH, as depicted in Figure 2A, In IH treated wells, EC-CFUs numbers decreased by 70% (from 16.4 ± 7 EC-CFUs/well without NAC to 4.7 ± 3 with NAC, *p* < 0.008). Treatment with SH, decreased EC-CFUs numbers by about 35% (from 9.2 ± 5.6 EC-CFUs/well without NAC to 5.9 ± 4.1 with NAC, *p* < 0.008). Also in normoxia the number of colonies was decreased by about 45% in NAC treated cells (from 7.7 ± 4.2 EC-CFUs/well to 4.2 ± 2.4, with NAC, *p* < 0.008).

### The effects of intermittent hypoxia and sustained hypoxia on EC-CFUs area

Interestingly, assessing the effects of Norm, IH and SH on the area of each of the EC-CFUs which developed in the above cultures revealed no significant differences between the various oxygen treatments. Figure 2B presents the mean area of EC-CFUs under IH, SH and normoxia. Also, the area of EC-CFUs was not significantly affected by treatment with NAC in either of the oxygen conditions. The area of each colony was measured using ImageJ program. Representative photomicrographs of EC-CFUs treated by normoxia, IH or SH on day 7 in culture as specified in Methods, are depicted in Figure 2C.

### ROS production is increased in intermittent and sustained hypoxia treated EC-CFUs

**Figure 3:**
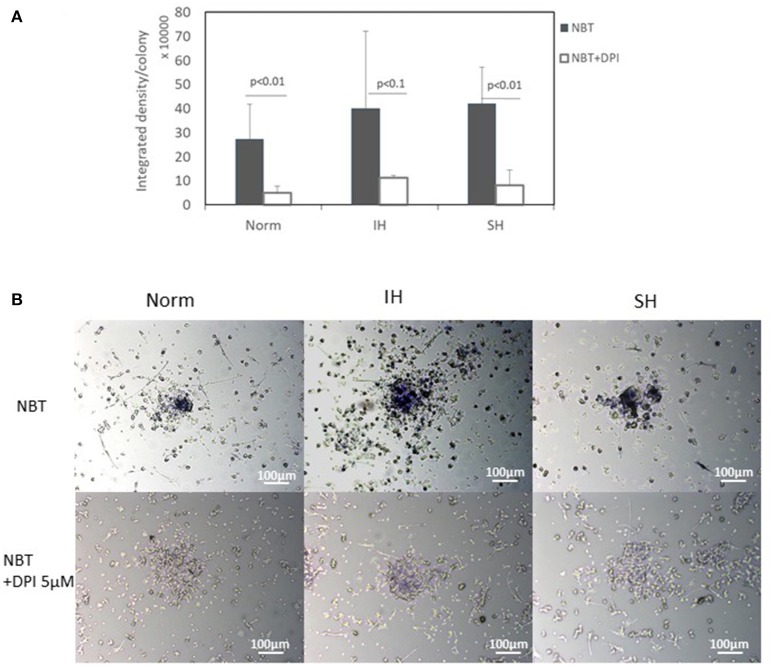
Production of Reactive oxygen species (ROS) by EC-CFUs under normoxia, intermittent and sustained hypoxia with and without diphenyl iodide (DPI). Production of ROS per colony was measured by the nitro blue tetrazolium (NBT) test with and without the NADPH oxidase inhibitor DPI (5 μM) (*n* = 3). The test depends on the direct reduction of NBT into an insoluble blue formazan compound by NADPH oxidase. Cytoplasmic clumps of formazan deposits are considered positive for ROS. The blue score positively correlates with cellular ROS production. **(A)** EC-CFU formazan optic density on the 7th day in culture with and without DPI under normoxia (Norm), intermittent hypoxia (IH) and sustained hypoxia (SH) (*p* < 0.01 for: Norm vs. Norm+DPI, SH vs. SH+DPI; *p* < 0.1 for IH vs. IH+DPI). **(B)** Representative photomicrographs of EC-CFUs microscopic fields (X10) of NBT test for Norm, IH and SH with and without 5 μM DPI.

ROS production was determined in 7-day EC-CFUs cultures by the Nitro Blue Tetrazolium (NBT) test (*n* = 3), which positively correlates with cellular ROS production. EC-CFUs cultures were exposed to Norm, IH and SH, with and without the NADPH oxidase inhibitor diphenyl iodide (DPI) (5 μM) as specified in Methods. As presented in Figures 3A,B the production of ROS is evident in EC-CFUs cultured under all three oxygen conditions. ROS production per colony was slightly elevated in IH treated colonies as compared to Norm (statistically not significant) and was largely attenuated by DPI (Norm vs. Norm + DPI, *p* < 0.01; IH vs. IH + DPI, *p* < 0.1; SH vs. SH + DPI, *p* < 0.01). The *P*-value < 0.1 obtained in IH vs. IH+DPI likely resulted from the large standard deviation between subjects.

### Protein carbonyl formation is increased in intermittent hypoxia treated EC-CFUs cultures

**Figure 4:**
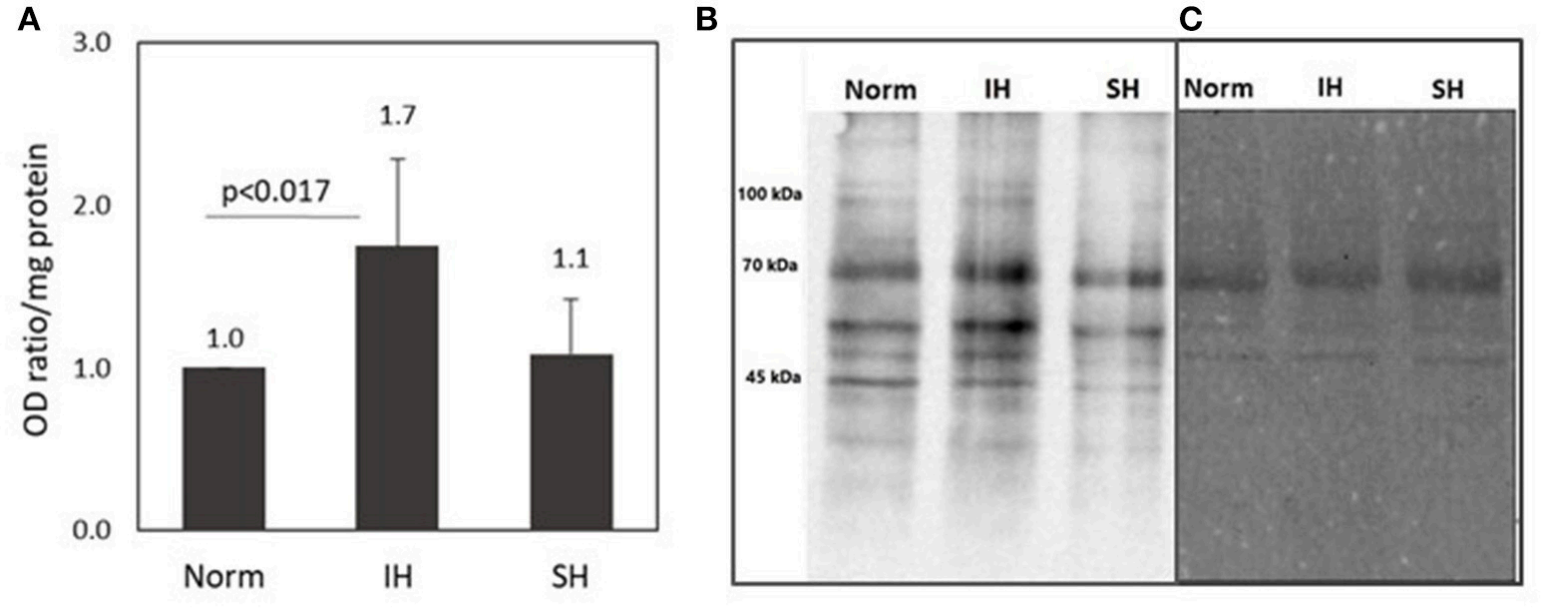
Protein carbonyl formation in EC-CFUs cultures exposed to normoxia, intermittent and sustained hypoxia. Analysis of protein carbonyls levels was based upon 2,4-dinitrophenylhydrazine (DNPH) carbonyl derivation, following immunodetection by using Western Blot (WB) assay with anti-dinitrophenol (DNP) antibodies, followed by densitometric quantitation. **(A)** Average densitometric quantitation of WB assays of EC-CFUs cultures from 4 different donors exposed to normoxia (Norm), intermittent hypoxia (IH) and sustained hypoxia (SH). (*p* < 0.017 for: Norm vs. IH) **(B)** A representative WB analysis of total intracellular protein carbonyls from EC-CFUs cultures in Norm, IH, and SH. **(C)** The control corresponding Ponceau S total protein stain of the membrane.

The protein carbonyl assay is an indicator of protein oxidative modification. As such, it was used to identify carbonylated proteins in EC-CFUs cultures exposed to Norm, IH and SH. Protein carbonyl levels were analyzed using commercially available OxyBlot Protein Oxidation Detection Kit (Millipore, USA), based upon 2, 4-dinitrophenylhydrazine (DNPH) carbonyl derivation, following immunodetection. Carbonylated proteins were immunodetected by Western Blotting using anti-dinitrophenol (DNP) antibodies, and consequently analyzed by densitometric quantitation. As shown in Figure 4, protein carbonylaion per mg protein was 1.7-fold higher under IH as compared to normoxia (*p* < 0.017). Representative photomicrographs of a western blot of carbonylated proteins and their protein determination by Ponceau S protein stain are depicted in Figures 4B,C.

### The potential involvement of NADPH oxidase on EC-CFUs formation

**Figure 5:**
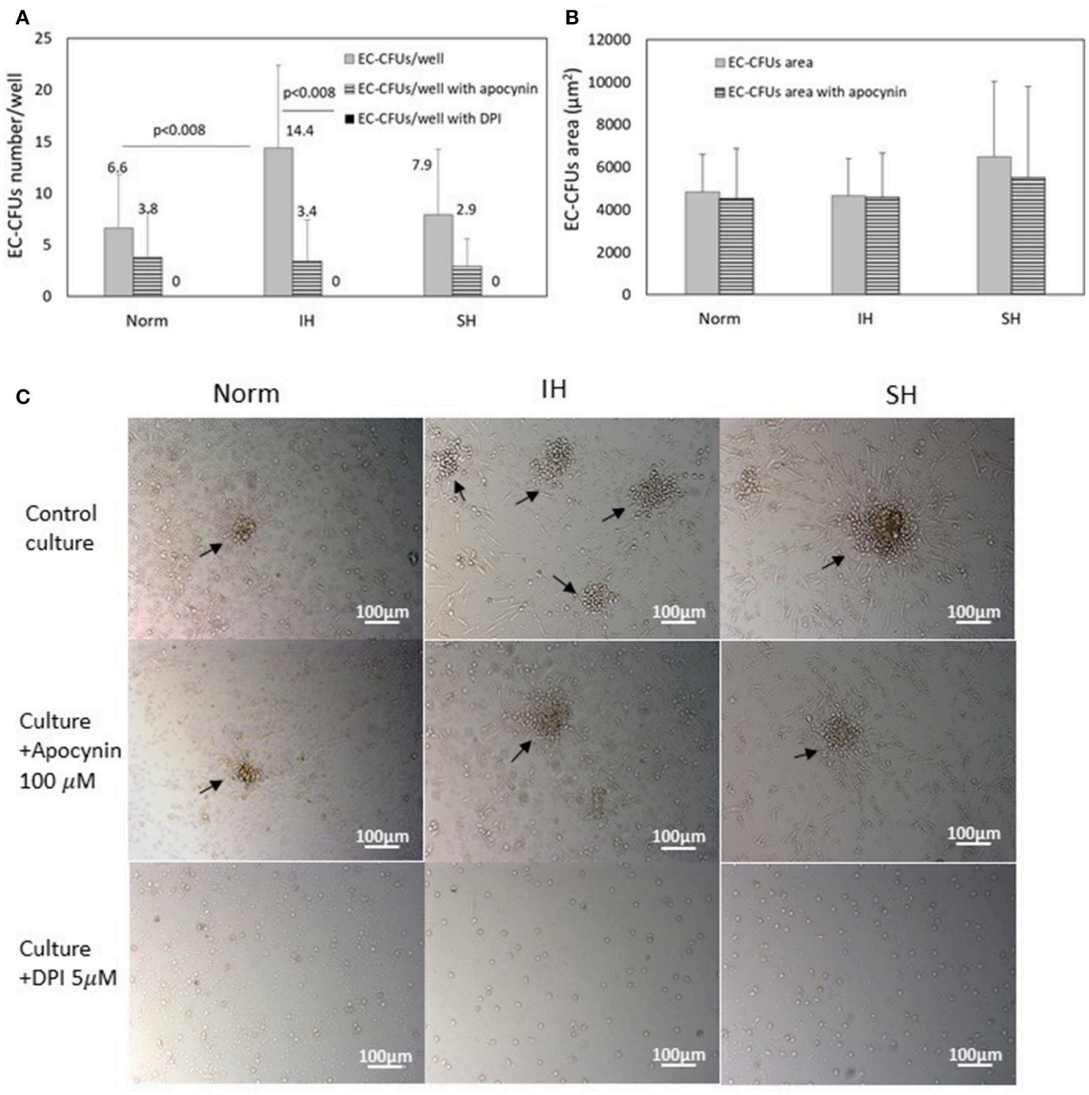
The effects of intermittent and sustained hypoxia *in vitro* on EC-CFUs number and area with and without treatment with apocynin or diphenyl iodide (DPI). **(A)** Mean EC-CFUs numbers on the 7th day in culture with and without apocynin (100 μM) or diphenyl iodide (DPI) (5 μM) (*n* = 8). Control cells were exposed for 3 days to intermittent hypoxia (IH) and sustained hypoxia (SH) as specified in Methods and compared to normoxia (Norm). Additional cell cultures were exposed to apocynin or DPI concomitantly with Norm, IH and SH. (*p* < 0.008 for: Norm vs. IH, IH vs. IH+apocynin). Following DPI treatment no cellular maturation and no EC-CFUs formation was noted. **(B)** Mean EC-CFU size on the 7th day in culture with and without apocynin (*n* = 8) was unaffected. No significant differences were found between the areas of EC-CFUs treated with various oxygen treatments or with apocynin. **(C)** Representative photomicrographs of EC-CFUs microscopic fields (X10) for Norm, IH and SH with and without apocynin or DPI. EC-CFUs colonies are indicated by arrows.

In order to determine the potential involvement of NADPH oxidase-dependent ROS on the development of EC-CFUs numbers under Norm, IH and SH, two distinct NADPH oxidase inhibitors were utilized, i.e., apocynin (100 μM) and DPI (5 μM), (*n* = 8). As depicted in Figure 5A, the mean number of colonies was significantly lower in apocynin treated cultures. The most dramatic effect was noted in the IH-treated cells, in which, a 76% decrease in EC-CFUs numbers per well was noted (3.4 ± 4.1 EC-CFUs/well with apocynin compared to 14.4 ± 8 without apocynin, *p* < 0.008). The number of EC-CFUs per well was also lowered in Norm and apocynin treated cultures by about 43% (3.8 ± 4.5 EC-CFUs/well with compared to 6.6 ± 5.2 without apocynin), but these values were not statistically significant. A similar trend was also noted in SH-treated cultures, apocynin lowered 63% of the EC-CFUs per well. However, addition of DPI completely abolished EC-CFUs development and formation in all oxygen conditions. The EC-CFUs area was also assessed in the presence of apocynin, as depicted in Figure 5B. The mean EC-CFUs area was not significantly affected in either of the oxygen treatments. Representative photomicrographs of EC-CFUs cultures with and without the NADPH oxidase inhibitors apocynin and DPI are depicted in Figure 5C.

### Effects of intermittent hypoxia, NAC and NADPH oxidase inhibitors on Gp91-phox subunit expression in EC-CFUs

**Figure 6:**
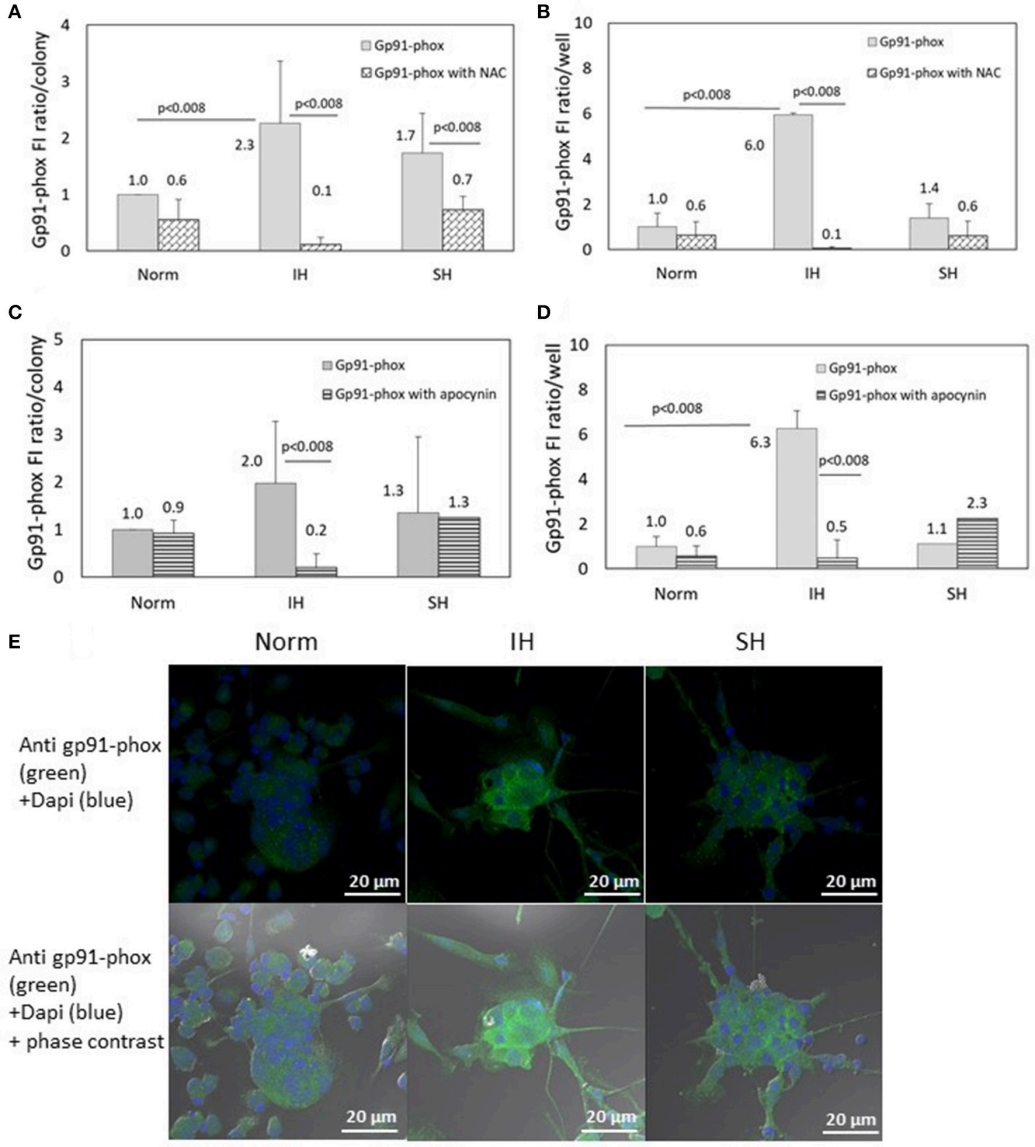
The effects of normoxia, intermittent and sustained hypoxia on the expression of NADPH oxidase gp91-phox subunit in EC-CFUs in the presence of N-acetylcysteine (NAC) and apocynin. Gp91-phox specific fluorescence intensity (FI) was detected using confocal microscopy in EC-CFUs cultured under normoxia (Norm), intermittent hypoxia (IH) and sustained hypoxia (SH). Fixed cells were stained with rabbit anti- gp91-phox primary Abs (diluted 1/150) followed by 1/400 CF 488A anti-rabbit IgG staining (green). Nuclei were stained with DAPI (blue). **(A)** Average specific FI of gp91-phox subunit expression per colony, with and without 1 mM NAC in (*p* < 0.008 for: IH vs. Norm, IH vs. IH+NAC and SH vs. SH+NAC). **(B)** Average FI of specific gp91-phox subunit expression in EC-CFUs per culture well with and without the addition of 1 mM NAC, (*p* < 0.008 IH vs. Norm, IH vs. IH+NAC). **(C)** Average FI of gp91-phox subunit specific expression per colony, with and without the addition of 100 μM apocynin, (*p* < 0.008 IH vs. IH+apocynin). **(D)** Average FI of specific gp91-phox expression in EC-CFUs per culture well with and without the addition of 100 μM apocynin, (*p* < 0.008 IH vs. Norm, IH vs. IH+apocynin). **(E)** Representative photomicrographs of gp91-phox subunit expression in each of the EC-CFUs treated under Norm, IH and SH (X40). In **(A–D)** determination in three independent experiments.

The specific fluorescence intensity (FI) of Gp91-phox was detected using confocal microscopy. EC-CFUs cultured under Norm, IH and SH with and without NAC (1 mM) or apocynin (100 μM) were referenced to fold increase from normoxia. The FI ratio of Gp91-phox per colony was elevated by 2.3- and 1.7-fold under IH and SH, respectively (Figure 6A, *p* < 0.008 IH vs. Norm). The FI ratio of Gp91-phox per well was elevated by 6-fold under IH compared to Norm (*p* < 0.008), probably due to the higher EC-CFUs numbers per well (Figure 6B). Addition of NAC (1 mM) significantly decreased gp91-phox expression in the IH and SH-treated colonies, in which the expression was elevated. Total expression per well was also decreased in the presence of NAC (Figure 6B). Also apocynin, drastically decreased the FI ratio of the IH-treated cells, both per colony and per well but it did not decrease the FI ratios of Gp91-phox in normoxia and SH (Figures 6C,D). Representative photomicrographs of gp91-phox expression in EC-CFUs are depicted in Figure 6E.

### Effects of intermittent hypoxia on p22-phox subunit expression in EC-CFUs

**Figure 7:**
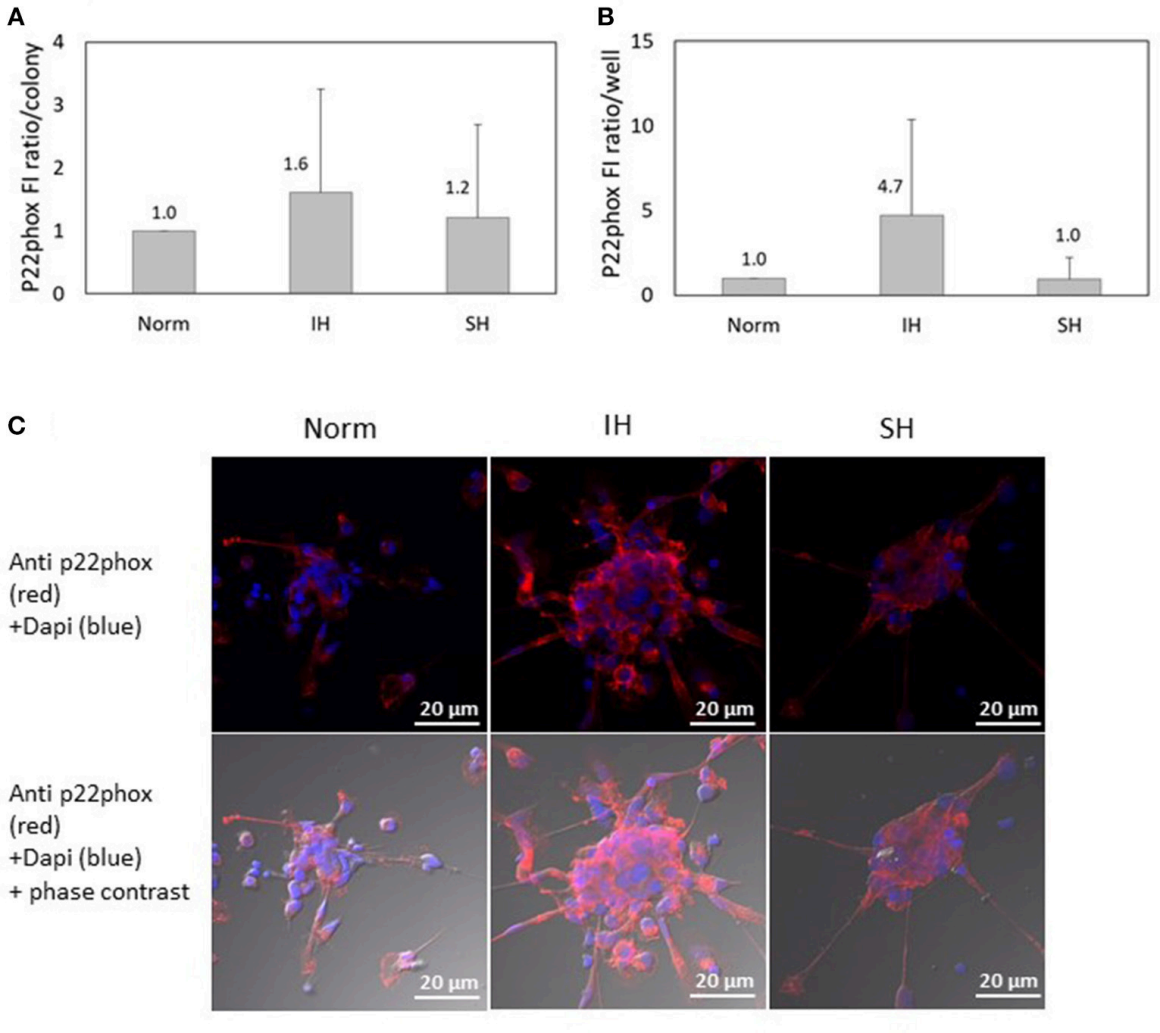
The effects of normoxia, intermittent and sustained hypoxia on the expression of NADPH oxidase p22phox subunit in EC-CFUs. p22phox specific fluorescence intensity (FI) was detected using confocal microscopy in EC-CFUs cultured under normoxia (Norm), intermittent hypoxia (IH) and sustained hypoxia (SH). Fixed cells were stained with mouse anti-p22phox primary Abs (diluted 1/100) followed by 1/400 CF 647 anti-mouse staining (red). Nuclei were stained with DAPI (blue), in 3 independent experiments. **(A)** Average FI of p22phox subunit specific expression per colony. **(B)** Average FI of specific p22phox subunit expression in EC-CFUs per culture well. **(C)** Representative photomicrographs of p22phox subunit expression in each of the EC-CFUs treated under Norm, IH and SH (X40).

Another NADPH oxidase subunit investigated, p22-phox, was also detected in EC-CFUs cultured under Norm, IH and SH using confocal microscopy. As shown in Figure 7A, the specific FI ratio of p22-phox per colony was slightly elevated by 1.6- and 1.2-fold under IH and SH, respectively, as compared to EC-CFUs cultured under normoxia, (both not statistically significant). As expected, the expression of p22-phox per well was elevated by 4.7-fold in response to IH as compared to normoxia, likely, due to the higher EC-CFUs numbers per well (Figure 7B) Yet, these differences did not reach a statistical significance. Representative photomicrographs of p22-phox expression in EC-CFUs are depicted in Figure 7C.

### Nuclear factor-erythroid 2 related factor 2 (Nrf2) is expressed in EC-CFUs, but not affected by IH

**Figure 8:**
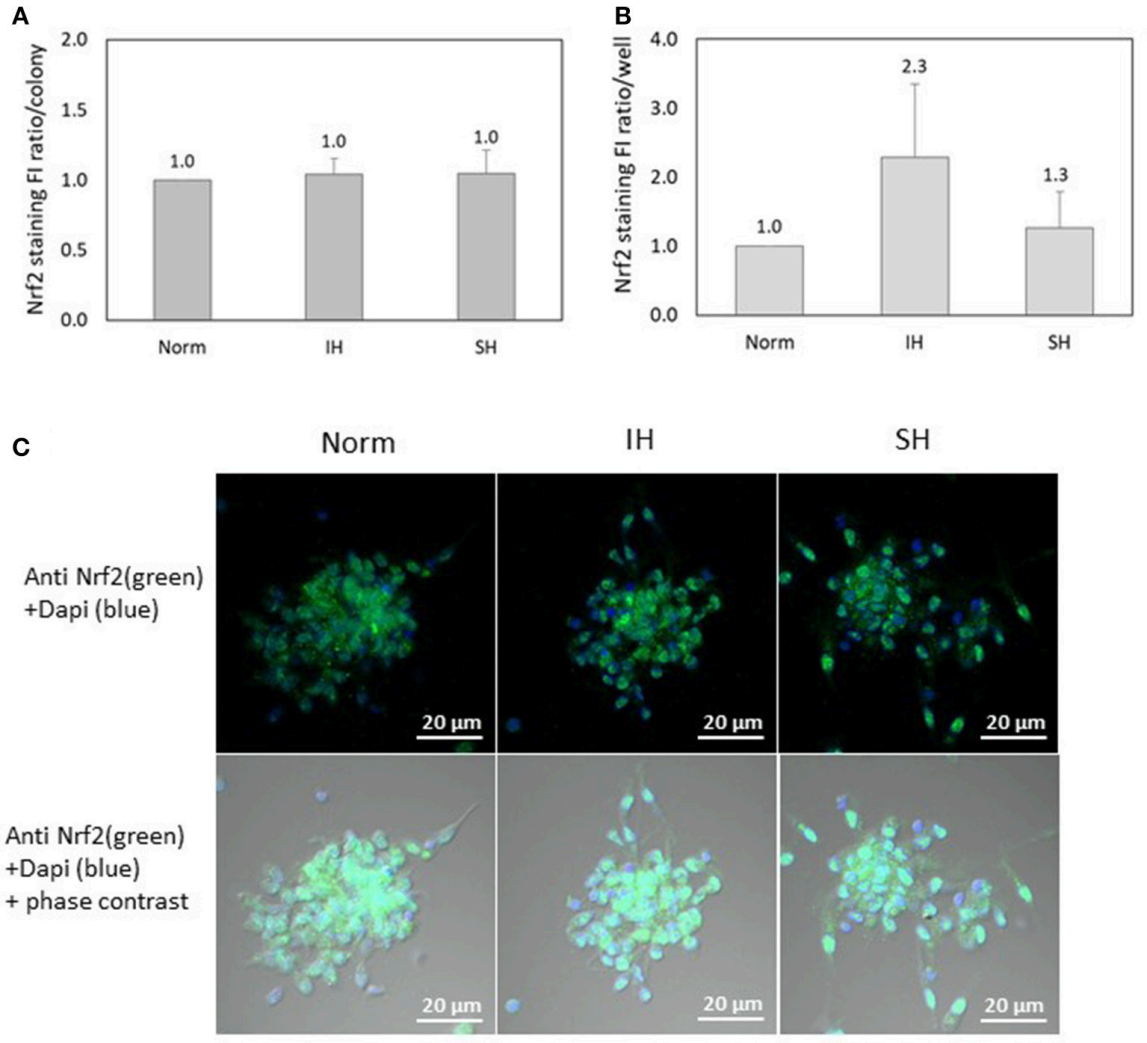
The effects of normoxia, intermittent and sustained hypoxia on Nuclear factor-erythroid 2 related factor 2 (Nrf2) expression in EC-CFUs. Nrf2 specific fluorescence intensity (FI) was detected using confocal microscopy in EC-CFUs cultured under normoxia (Norm), intermittent hypoxia (IH) and sustained hypoxia (SH). Fixed cells were stained with rabbit anti-Nrf2 primary Abs (diluted 1/150) followed by 1/400 CF 488A anti-rabbit IgG staining (green). Nuclei were stained with DAPI (blue). **(A)** Average FI of Nrf2 specific expression per colony under Norm, IH and SH. **(B)** Average FI of specific Nrf2 expression in EC-CFUs per well under to Norm, IH and SH. In **(A,B)**, integrated with Image J Software, in 3 independent experiments. **(C)** Representative photomicrographs of Nrf2 expression in each of the EC-CFUs treated under Norm, IH and SH (X40).

Nrf2 specific FI was detected using confocal microscopy in EC-CFUs cultured under Norm, IH and SH and quantified by densitometry (*n* = 3). As depicted in Figure 8A, Nrf2 expression per colony was unaffected by IH or SH compared to normoxia. However, the integrated FI of specific Nrf2 expression per well was non-significantly increased by 2.3-fold as compared to normoxia (Figure 8B). This indicates that FI of Nrf2 per colony is the same at all treatment conditions, while the increase observed under IH is due to the higher numbers of colonies per well. Representative photomicrographs of Nrf2 specific fluorescence in EC-CFUs are shown in Figure 8C.

### EC-CFUs cell proliferation under intermittent and sustained hypoxia

**Figure 9:**
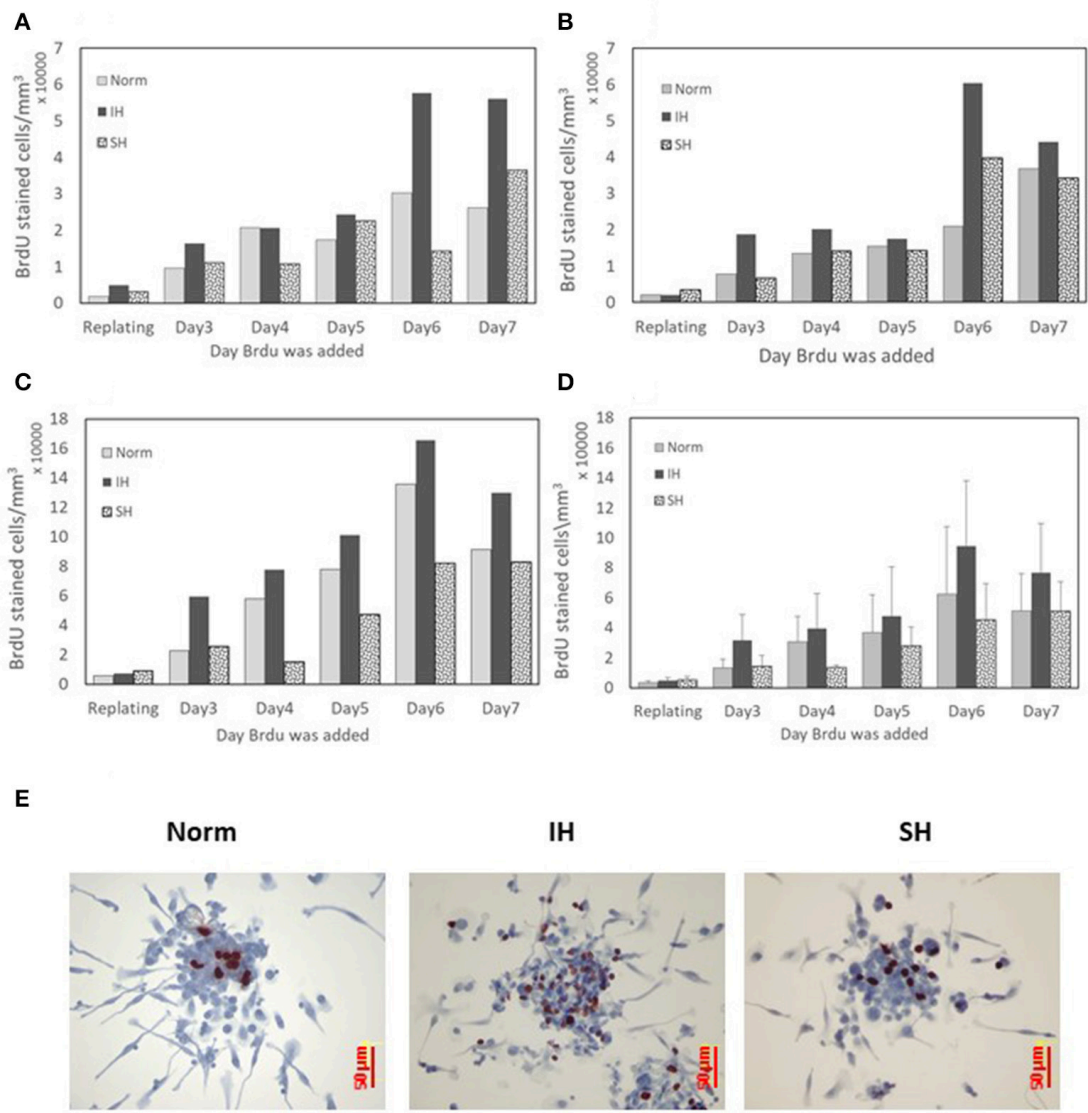
The effects of normoxia, intermittent and sustained hypoxia on cellular proliferation in EC-CFUs. Cellular proliferation was detected by pulse labeling colonies with bromodeoxyuridine (BrdU). Cells were cultured under normoxia (Norm), intermittent hypoxia (IH) and sustained hypoxia (SH) from the re-plating time until day 7 in culture for 6 h every day. **(A–C)** BrdU labeled cells in the developing EC-CFUs in culture on days 3–7. Each figure represents individual data from a blood donor. **(D)** Denotes the average values of BrdU labeled cells ± SE in the developing EC-CFUs in culture on days 3–7 for the three subjects. **(E)** Representative photomicrographs (x40) demonstrating the incorporation of BrdU (dark brown) into proliferating cells within each of the EC-CFUs treated under Norm, IH, and SH (on day 6).

The proliferative capacity of EC-CFUs formation in culture was determined by bromodeoxyuridine (BrdU) uptake. A significant BrdU uptake was noted at all oxygen conditions. The results from 3 independent experiments performed with cultures from 3 donors are presented for each subject separately in Figures 9A–C, while Figure 9D depicts the average data for these subjects. The concentration of BrdU stained cells in each well gradually increased from day 3 to 7 in culture and was mostly notable on day 6 in culture, particularly in the IH treated cells. However, these differences did not reach a statistical significance. Depicting each donor's data individually highlights this repeated pattern for all oxygen treatments. EC-CFUs proliferation under SH was similar or slightly lower than in normoxia. As in previous experiments, the colony area was relatively unaffected regardless of the oxygen treatment applied, while the increased EC-CFUs numbers per well under IH contributed to the increased BrdU incorporation per well, as illustrated in Figures 9A–D. Figure 9E represents photomicrographs depicting the incorporation of BrdU into proliferating cells within a colony under normoxia, IH and SH on day 6 in culture.

### Paracrine effects of intermittent and sustained hypoxia treated EC-CFUs on endothelial tube formation

**Figure 10:**
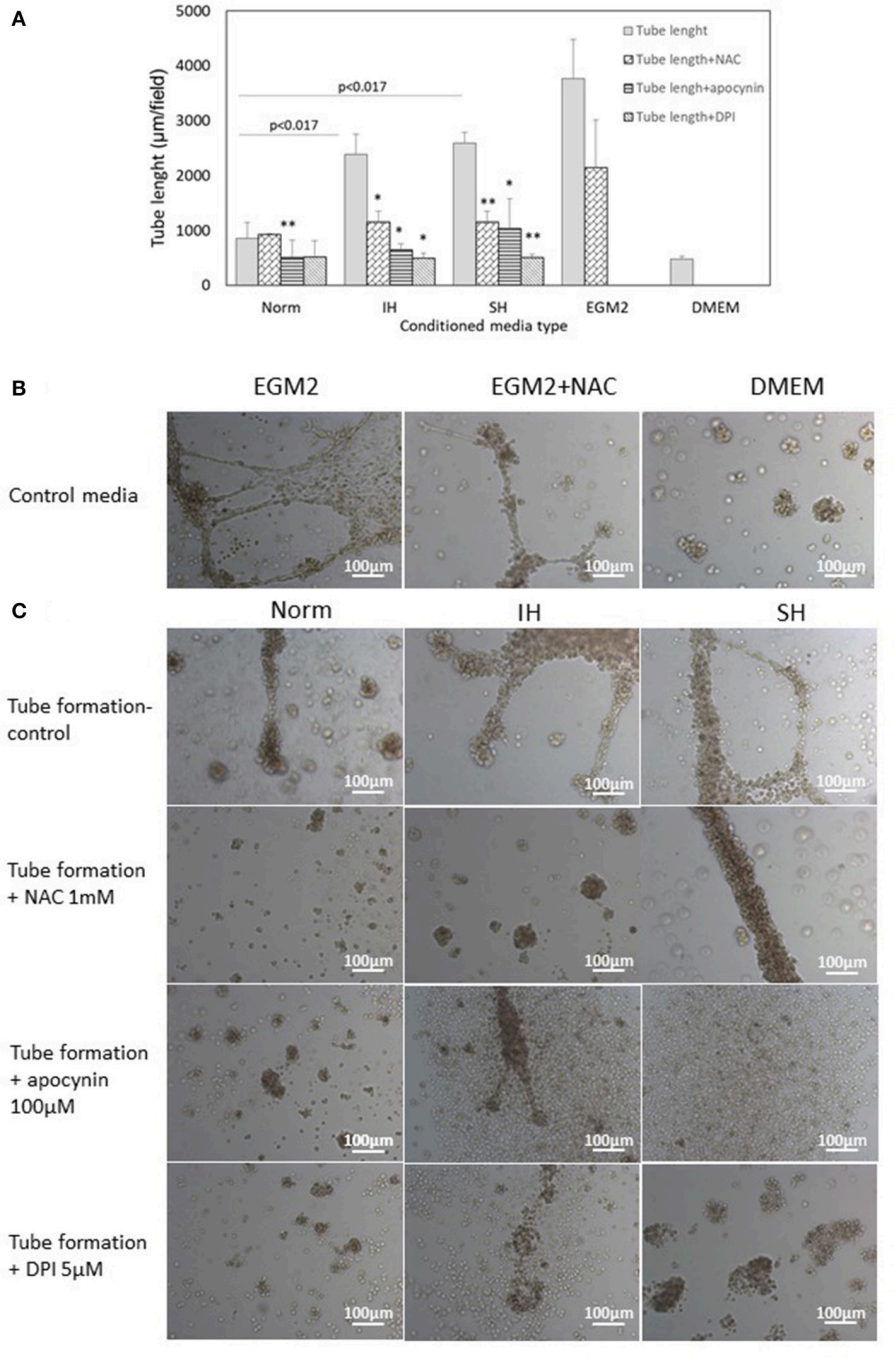
Mean endothelial tube length following treatment with conditioned media harvested from normoxia, intermittent and sustained hypoxia treated EC-CFUs with and without ROS inhibitors. **(A)** Tube formation by EAhy926 endothelial cells grown for 24 hrs on ECM-Gel with conditioned media harvested from normoxia (Norm), intermittent hypoxia (IH) and sustained hypoxia (SH) treated EC-CFUs (*n* = 3). Inhibitors used: 1 mM N-acetylcysteine (NAC), 100 μM apocynin and 5 μM diphenyl iodide (DPI). For a positive control, EAhy926 endothelial cells were grown with EGM-2 medium supplemented 20% FCS, and for a negative control, DMEM medium supplemented with 20% FCS was used (*p* < 0.017 for IH and SH vs. Norm, ^*^p < 0.05, ^**^p < 0.01 compared to Norm in each condition group), as specified in Materials and Methods **(B)** Representative photomicrographs (X10) of endothelial tube formation with EGM-2 without and with 1 mM NAC, and with DMEM. **(C)** Representative photomicrographs (X10) of endothelial tube formation with Norm, IH and SH conditioned media using the specified inhibitors.

**Figure 11:**
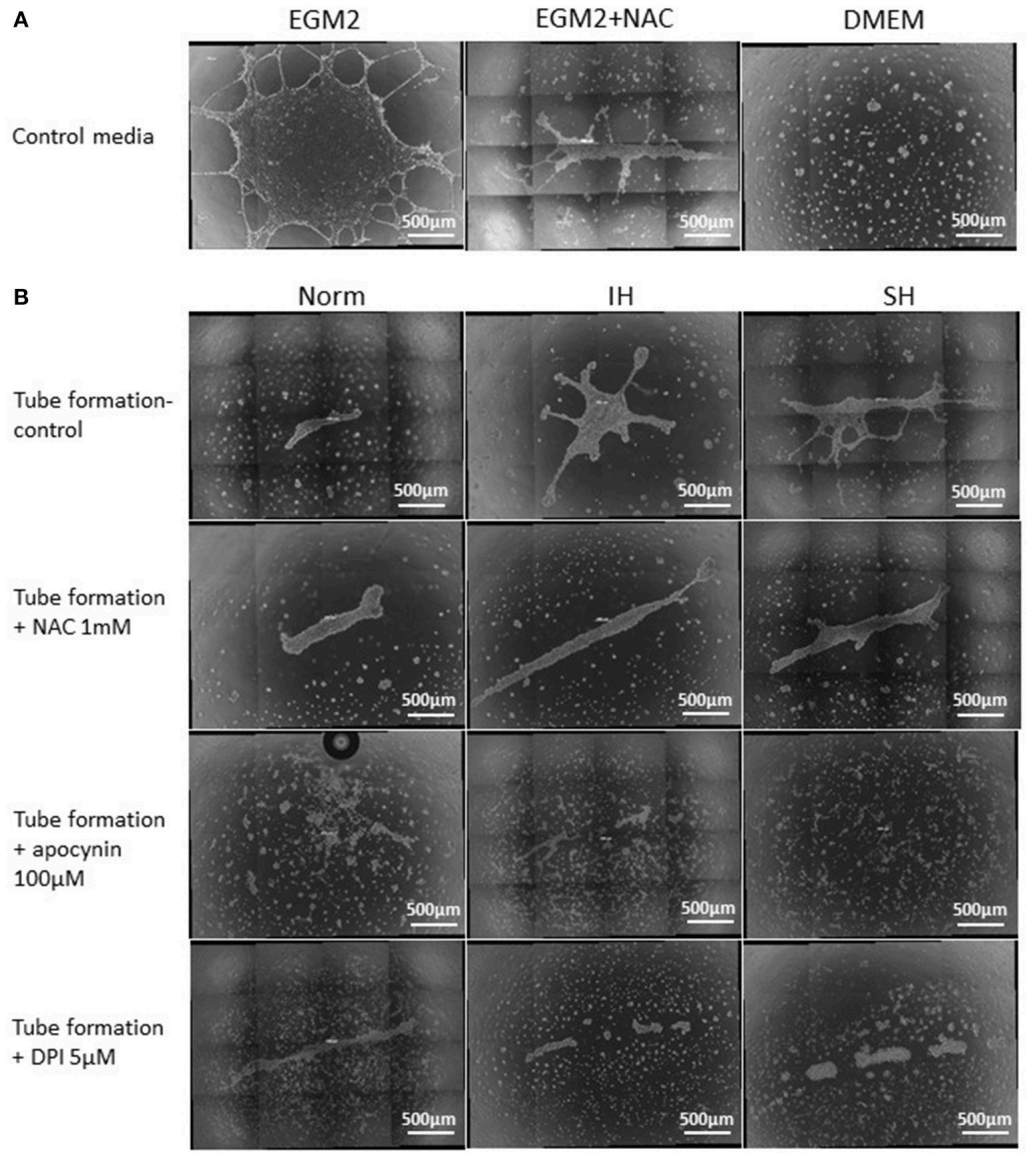
Whole well images of endothelial tube formation by EAhy926 endothelial cells following treatment with EC-CFUs conditioned media from normoxia, intermittent and sustained hypoxia using various inhibitors. **(A)** Whole well tube formation by EAhy926 endothelial cells grown for 24 hrs on ECM-Gel with EGM-2 medium without and with NAC, and with DMEM medium (as specified in Figure 10). **(B)** Whole well tube formation by EAhy926 endothelial cells grown for 24 hrs on ECM-Gel with conditioned media harvested from normoxia (Norm), intermittent hypoxia (IH) and sustained hypoxia (SH) treated EC-CFUs. Inhibitors included 1 mM N-acetylcysteine (NAC), 100 μM apocynin and 5 μM diphenyl iodide (DPI). In **(A,B)**, High-resolution whole-well imaging at x10 magnification with post-acquisition automatic image stitch were performed.

Endothelial tube formation is an *in vitro* assay indicative of vascular function. Endothelial cells from the EA.hy.296 cell line were seeded on extracellular matrix coated plates in the presence of conditioned media obtained from EC-CFUs cultures which developed under Norm, IH and SH. Generation of networks of vessel-like structures was determined by calculating their net length in culture wells as described in Materials and Methods. Complete endothelial growth medium EGM-2 supplemented with 20% FCS served as a positive control for tube formation. DMEM medium supplemented with 20% FCS served as a negative control (Figures 10A,B). As expected, tube formation was not observed in DMEM treated EA.hy.296 cells. Additionally, tube formation was also performed with and without NAC (1 mM), apocynin (100 μM) and DPI (5 μM). As depicted in Figures 10A,C, treatment with conditioned media obtained from IH and SH-treated cells significantly increased endothelial tube formation as compared to normoxia (2388 ± 359 and 2,586 ± 196 vs. 855 ± 287 μm/field, respectively, *p* < 0.0017 for both). Adding NAC or DPI to IH- and SH-treated cells significantly decreased tube length formation, respectively (both *p* < 0.05). Also apocynin significantly decreased tube length formation as compared to all conditioned media (for Norm, IH and SH, *p* < 0.05). Representative photomicrographs of endothelial tube formation with EC-CFUs conditioned media from Norm, IH and SH with and without the addition of inhibitors NAC, DPI and apocynin are shown in Figure 10C. Whole-well images of endothelial tube formation with EC-CFUs conditioned media from the three oxygen conditions with and without the addition of inhibitors or growth factors are shown in Figure 11.

### VEGF specific fluorescence is affected by IH and SH

**Figure 12:**
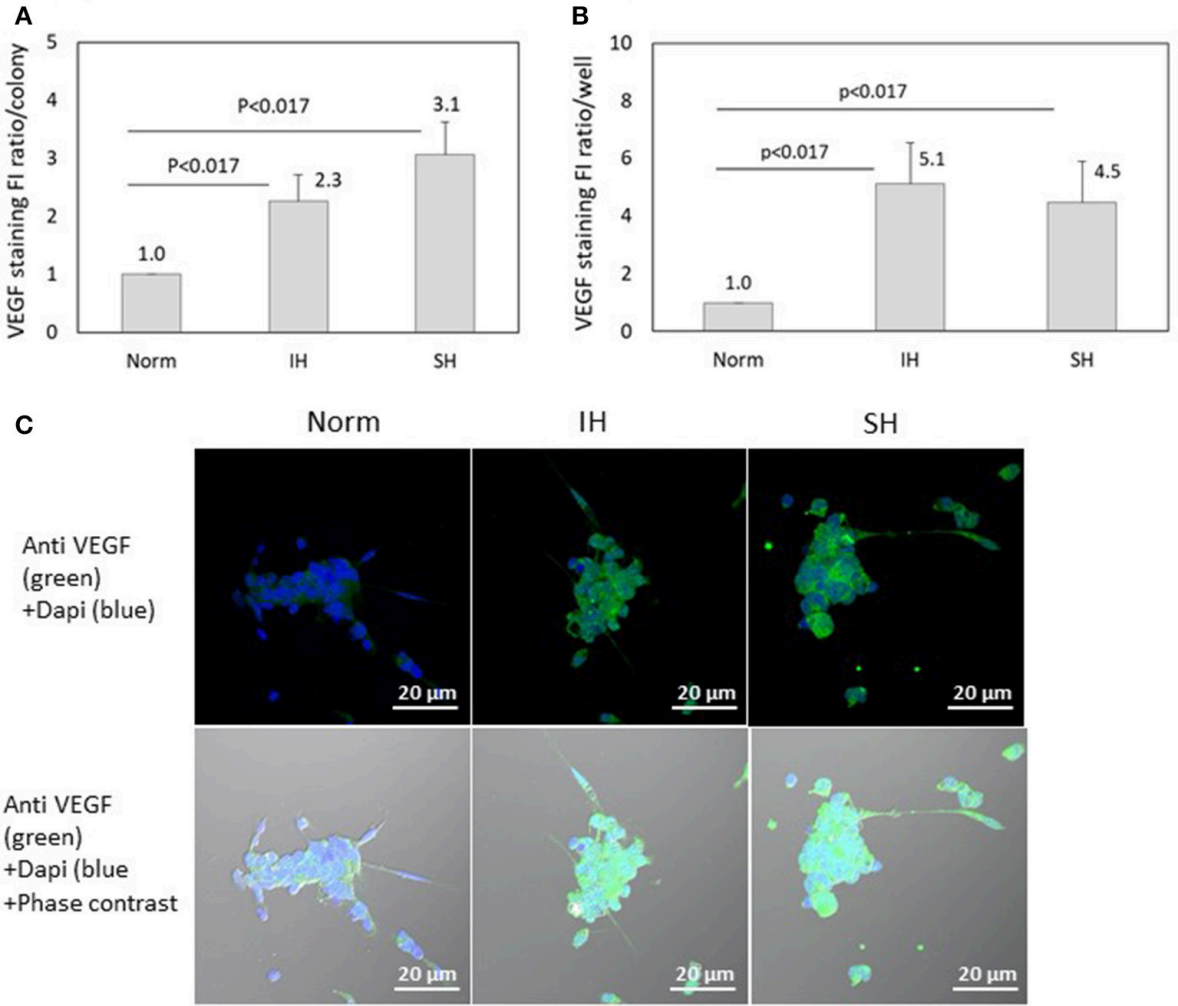
The effects of normoxia, intermittent and sustained hypoxia on VEGF expression in EC-CFUs. VEGF specific fluorescence intensity (FI) was detected using confocal microscopy in EC-CFUs cultured under normoxia (Norm), intermittent hypoxia (IH) and sustained hypoxia (SH). Fixed cells were stained with rabbit anti-VEGF primary Abs (diluted 1/250) followed by 1/400 CF 488A anti-rabbit IgG staining (green). Nuclei were stained with DAPI (blue). **(A)** Average FI of VEGF specific expression per colony, integrated with Image J Software in 4 independent experiments (*p* < 0.017 IH vs. Norm, SH vs. Norm). **(B)** Average FI of specific VEGF expression in EC-CFUs in a whole culture well integrated with Image J Software, in 4 independent experiments (*p* < 0.017 IH vs. Norm, SH vs. Norm). **(C)** Representative photomicrographs of VEGF expression in each of the EC-CFUs treated under Norm, IH and SH (X40).

VEGF specific FI was detected using confocal microscopy in EC-CFUs cultured under Norm, IH and SH. The ratio of VEGF specific FI per colony was elevated by 2.3- and 3.1-fold under IH and SH, respectively (*p* < 0.017 for both), as compared to normoxia (Figure 12A). However, the expression of VEGF per well was increased by 5.1- and 4.5-fold under IH and SH, respectively, as compared to normoxia (Figure 12B). Representative photomicrographs of VEGF expression in EC-CFUs are depicted in Figure 12C.

## Discussion

The deleterious effects of IH and OSA on the cardiovascular system are well established. Many of which, result from the IH-associated oxidative stress and systemic inflammation. Yet, stimulation of adaptive-protective mechanisms reminiscent of ischemic preconditioning, is also associated with IH and OSA and might play an essential role in cardiovascular health (4).

Development of EC-CFUs in culture is an acceptable surrogate marker representing vasculoprotective mechanisms. EC-CFUs are reduced in patients with overt atherosclerosis, are diminished in chronic cardiovascular disease and contribute to vascular repair involving paracrine functions (7, 10, 11, 18). In the current study, the increased formation of EC-CFUs colonies which developed under IH *in vitro* is consistent with a previous report from our laboratory describing this phenomenon in patients with AMI and concomitant SDB as well (12). However, the significance of ROS to their development is described here for the first time. Importantly, although colony formation was elevated in IH-treated cells in all donors, the number of EC-CFUs generated varied considerably between subjects. Moreover, subjects investigated on two to three different experiments displayed reproducible donor individual responses. Thus, donors who had a higher capacity to develop EC-CFUs under normoxia, also developed higher EC-CFUs numbers under IH *in vitro*. Individual variations in responses to hypoxia or intermittent hypoxia are not uncommon in human subjects. For instance, cells or tissues from different individuals that were exposed to the same hypoxic stimulus had diverse responses in hypoxia-sensitive transcription factors, downstream gene products, and transduction pathways (26–28). This was shown for VEGF and other downstream genes of HIF-1α, suggesting that the source of this variation resides within the HIF system itself (26). Such inter-individual differences were also exemplified in an earlier study reporting on a positive and highly significant correlation between the VEGF response to hypoxia in monocytes harvested from coronary artery disease patients, and the presence of collaterals in the heart (27). These inter-individual differences are further exemplified by demonstrating that HIF polymorphism was associated with the development of collaterals in patients with ischemic heart disease, suggesting that variations in the HIF-1α genotype may affect the development of coronary artery collaterals in patients with significant coronary artery disease (28). Such heterogenic responses to a given hypoxic stimulus due to genetic variations may explain the variable angiogenic responses between individuals to a given hypoxic stimulus. In view of the putative role of EC-CFUs in cardiovascular health, the large inter-individual variability expressed in EC-CFUs number and function to a given IH stimulus may represent an individual trait with a potential clinical significance. This is consistent with the ability of patients with coronary artery disease exhibiting increased or decreased EPCs numbers, to develop adequate or inadequate coronary collaterals congruently with collateral flow index, EC-CFUs numbers and tube formation (16, 17, 29).

As mentioned earlier, AMI patients with concomitant SDB, developed higher EC-CFUs numbers and their paracrine-angiogenic properties were shown to increase as compared to AMI patients without SDB. Since treatment with IH *in vitro* was also shown to increase the EC-CFUs numbers and angiogenic properties, these elevations were attributed to the IH-associated with SDB in these AMI patients (12). In accord with this line, in patients with coronary artery disease and concomitant OSA, coronary collateral development was improved as compared to patients with coronary artery disease without OSA (29–31). These finding are also reinforced by animal studies demonstrating that myocardial infarct size and ischemic brain injury were attenuated by exposure to IH (32, 33). It is also indicated that in AMI patients with concomitant SDB, despite having higher rates of co-morbidities, SDB was not associated with adverse clinical outcomes compared to AMI patients without SDB (34). Also, patients with OSA were shown to have less severe cardiac injury during an acute non-fatal MI (35). In patients with acute stroke and OSA, although higher rates of cardiovascular disease were noted, patients had less severe neurological injury and lower adjusted mortality rates (36) and better adjusted survival rates (37). Collectively, these finding support the existence of adaptive responses in OSA through IH as well. Likely, some IH patterns are more efficacious then others in promoting protective effects (38).

Oxidative stress resulting from IH or from OSA-associated IH is a fundamental mechanism, and was shown to result from increased ROS formation by various activated leukocytes subpopulations and endothelial cells (1, 5, 39). Similarly, also EC-CFUs exposed to IH produced higher amounts of ROS compared to normoxia, as indicated by the NBT test and by protein carbonylation, both well-established markers of ROS production and oxidative stress.

Of note, ROS-associated protein carbonylation may lead to protein dysfunction, yet, protein carbonylation also plays an important role in cell signaling (40, 41). The significant increases in protein carbonylation evident in EC-CFUs cultures exposed to IH were referenced per mg protein, demonstrating that oxidative stress affected proteins of IH-treated cells as compared to Normoxia or SH. Increased protein carbonylation was reported in other models of IH as well. For instance, in mice exposed to long-term IH, protein carbonylation as well as other oxidative stress markers were elevated in regions of the basal forebrain and brainstem (42). Similarly, the NBT test further supports increased ROS production by fully developed EC-CFUs treated by IH. This test is a diagnostic tool particularly for ROS-dependent leukocyte functions associated with NADPH oxidase, and positively correlates with proper NADPH oxidase function and ROS production. When performed on fully developed EC-CFUs, the NBT test showed ROS formation within the colonies under all oxygen conditions. Although, ROS formation was higher in IH-treated colonies it was not statistically significant. Addition of the potent NADPH oxidase inhibitor DPI, to fully developed EC-CFUs for the last IH and SH cycles, abolished ROS formation within these developed colonies under all oxygen conditions. However, when DPI was added at the re-plating stage, the formation of EC-CFUs and culture maturation was completely abolished. Of note, since DPI is also a potent inhibitor of cell growth and promotes apoptosis independently from ROS when used for longer periods, in such experiments we also compared the effects of apocynin which has been used as an effective low toxicity inhibitor of NADPH oxidase in many experimental models. Its mechanism of inhibition is not completely known but it lowers the levels of cytosolic p47phox subunit and impairs its translocation to the membrane, thus, blocking the assembly and activation of NADPH oxidase (43). Apocyanin allowed EC-CFUs formation, but attenuated the increases in their numbers in IH treated cells. However, once a colony was formed, the area of the developed EC-CFUs was not significantly affected by the addition of apocynin or NAC, basically maintaining a similar size at all oxygen conditions. This may indicate a built-in inherent colony structure, whose properties should be further explored. Collectively, these findings indicate that NADPH oxidase activity and ROS production contribute to the formation EC-CFUs. This is particularly evident under treatment with IH.

NADPH oxidase is known to be activated by various biological, chemical, physical and environmental factors. Hence it is suggested that NADPH oxidase and ROS-mediated signaling may be an important component of the cellular signal transduction network, inducing repair mechanisms (44). Along this line, NADPH oxidase was shown to facilitate erythropoietin signaling in EPCs, to promote hypoxia-induced mobilization and vascular repair in an animal model (45). Additionally, knocking down NADPH oxidase expression with siRNA significantly suppressed endothelial proliferation (46). This latter study supports the findings in the current study indicating that particularly under IH, NADPH oxidase facilitates EC-CFUs formation since inhibiting NADPH oxidase by apocynin decreased gp91-phox expression in these developing cultures. A similar effect was noted by adding NAC. However, its inhibitory effects on gp91-phox expression were noted in all oxygen treated cultures, possibly indicating that also scavenging ROS from other cellular sources such as mitochondria also affects NADPH oxidase expression. Thus, EC-CFUs formation is affected by ROS and functional NADPH-oxidase.

In parallel, in order to assess the antioxidant capacities in EC-CFUs, the expression of Nuclear factor-erythroid 2 related factor 2 (Nrf2) was determined. Nrf2 is a transcription factor regulating cellular detoxifying enzymes such as heme oxygenase-1 (HO-1) and phase II enzymes including glutathione-S-transferase (GST) and γ-glutamyl-transpeptidase (GGT) (47). It is constitutively expressed in almost all cell types including macrophages, but it is mainly expressed in tissues such as the liver, where routine detoxification reactions normally occur (48). Nrf2 was also found to have a vasculo-protective role in preventing pro-inflammatory signaling in endothelial cells (49). In the current study, Nrf2 was also expressed in EC-CFUs under all oxygen conditions applied, suggesting a constant need in antioxidant activity in these cells due to ROS formation. Yet, by exposing the cells to IH or SH, the expression of Nrf2 per colony was unaffected. Its levels were similar in the colonies that developed under all oxygen treatments, but the total expression of Nrf2 per well was elevated in IH-exposed cultures, due to the higher EC-CFUs numbers. Thus, the lack of induction of Nrf2 in the IH-treated colonies suggests that it does not have a significant role in altering the anti-oxidant ROS balance. Nonetheless, it may have additional anti-oxidant and anti-inflammatory roles in EC-CFUs that need to be further explored.

Previously, Padfield et al. (10) have shown that proliferation within the colony is required for EC-CFUs formation, but also migrating cells surrounding the colony had a minor contribution to colony development. In order to determine the contribution of the IH to colony development, the proliferative capacity within the colonies was investigated in all three oxygen conditions. Under normoxia the proliferative capacity was similar to earlier findings (10). The proliferative capacity of the SH treated colonies resembled that of normoxia, while BrdU incorporation in the IH treated colonies was slightly higher. This was particularly noted in each subject individually, but the averaged data did not reach statistical significance due to the large inter-individual differences. Likely, since the number of the IH-treated EC-CFUs was higher, also BrdU incorporation was higher in this treatment. Yet, the relative contribution of single cells recruited from the plate to colonies was not determined.

Finally, the paracrine effects of EC-CFUs were investigated by employing the endothelial tube formation assay. Endothelial cell monolayers were treated with conditioned media harvested from normoxia, IH and SH treated EC-CFUs cultures. Increased tube length was observed in wells grown with IH and SH conditioned media, indicating augmented paracrine angiogenic capacities under these conditions. The increased FI of VEGF expression per colony and per well in the IH and SH treated cells could at least partially explain the mechanism behind the increased tube length and paracrine capacity due to treatment with IH and SH conditioned media. The paracrine effects of VEGF from conditioned media are substantiated by an earlier study in which we have shown that tube formation was inhibited using anti-VEGF antibodies (bevacizumab) (19). Also adding NAC prevented the formation of vessel-like structures in Normoxia and IH, but attenuated tube formation with SH conditioned medium. These findings indicate that the presence of ROS in the medium is essential for promoting the vascularization abilities. The addition of apocynin as well as DPI also largely attenuated or abolished tube formation. Thus, the inhibition of NADPH oxidase and tube formation, particularly through apocynin, signify the role of NADPH oxidase in vascular function and endothelial tube formation through ROS as well. Apparently, at least two factors; VEGF and ROS participate in endothelial tube formation.

A limitation of this study should be acknowledged; the IH pattern employed in this *in vitro* study does not mimic the IH patterns observed in OSA patients. Despite these differences, the effects of IH *in vitro* on EC-CFUs numbers and functions were similar to those observed in our previous study investigating AMI patients with SDB. These IH *in vitro* patterns were also previously shown to affect leukocyte functions in a similar manner to that observed in OSA (4, 5, 24). Thus, IH promotes the development of EC-CFUs and their angiogenic properties. However, based on this *in vitro* study in healthy subjects, we cannot assess the potential contribution of these findings in the clinical setting.

## Conclusions

EC-CFUs were shown to closely correlate with vascular functions and retain a measure of vascular health. Evidently, IH contributed to increased EC-CFUs numbers and their properties such as ROS production and oxidative stress markers, VEGF expression and angiogenic capacities. Production of ROS and oxidative stress were shown to play an essential role in increasing EC-CFUs numbers and their angiogenic functions, as indicated by treatment with antioxidants such as NAC or NADPH oxidase inhibitors, which decreased these EC-CFUs capabilities. Although NADPH oxidase was shown to be an essential contributor of ROS, additional ROS sources could be involved as well, as indicated by NAC inhibition. Thus, IH *in vitro* was shown to activate adaptive mechanisms promoting the proliferative and angiogenic EC-CFUs properties. However, the ability to generate EC-CFUs was shown to be donor-dependent.

Collectively, these findings suggest that some IH patterns may provide cardio-protection in the context of OSA, possibly by inducing ischemic preconditioning. Yet, given the similarities in the findings obtained with IH *in vitro* and earlier findings on SDB patients with AMI, various IH patterns could be explored to identify the most efficacious IH patterns activating vascular adaptive-protective mechanisms in patients with OSA.

## Author contributions

LL, KA, and DA participated in the conceptual framework of the project. KA recruited the subjects and performed the experiments. KA, LL, and DA interpreted the data. KA and LL drafted the manuscript, and LL edited the manuscript. All authors approved the final version of the manuscript.

### Conflict of interest statement

The authors declare that the research was conducted in the absence of any commercial or financial relationships that could be construed as a potential conflict of interest.

## Abbreviations

IH: Intermittent hypoxia
SH: Sustained hypoxia
Norm: Normoxia
SDB: Sleep disordered breathing
OSA: Obstructive sleep apnea
EC-CFUs: Endothelial Cell-Colony Forming Units
ROS: Reactive oxygen species
NAC: N-acetylcysteine
DPI: Diphenyl iodide
EPCs: Endothelial progenitor cells
AMI: Acute myocardial infarction
NBT: Nitro blue tetrazolium
BrdU: Bromodeoxyuridine
Nrf2: Nuclear factor-erythroid 2 related factor 2
DNP: Dinitrophenyl
DNPH: Dinitrophenylhydrazine.
